# Synaptic Interactome Mining Reveals p140Cap as a New Hub for PSD Proteins Involved in Psychiatric and Neurological Disorders

**DOI:** 10.3389/fnmol.2017.00212

**Published:** 2017-06-30

**Authors:** Annalisa Alfieri, Oksana Sorokina, Annie Adrait, Costanza Angelini, Isabella Russo, Alessandro Morellato, Michela Matteoli, Elisabetta Menna, Elisabetta Boeri Erba, Colin McLean, J. Douglas Armstrong, Ugo Ala, Joseph D. Buxbaum, Alfredo Brusco, Yohann Couté, Silvia De Rubeis, Emilia Turco, Paola Defilippi

**Affiliations:** ^1^Department of Molecular Biotechnology and Health Sciences, Molecular Biotechnology Center, Università di TorinoTorino, Italy; ^2^The Institute for Adaptive and Neural Computation, School of Informatics, University of EdinburghEdinburgh, United Kingdom; ^3^Université Grenoble Alpes, iRTSV-BGEGrenoble, France; ^4^CEA, iRTSV-BGEGrenoble, France; ^5^Institut National de la Santé et de la Recherche Médicale, BGEGrenoble, France; ^6^Institute of Neuroscience, Consiglio Nazionale delle Ricerche (CNR)Milan, Italy; ^7^Humanitas Clinical and Research Center, IRCCSRozzano, Italy; ^8^Institut de Biologie Structurale, Université Grenoble AlpesGrenoble, France; ^9^CEA, DSV, IBSGrenoble, France; ^10^Centre National de la Recherche Scientifique, IBSGrenoble, France; ^11^GenoBiToUS-Genomics and Bioinformatics, Università di TorinoTurin, Italy; ^12^Seaver Autism Center for Research and Treatment, Department of Psychiatry, Icahn School of Medicine at Mount SinaiNew York, NY, United States; ^13^Department of Psychiatry, Icahn School of Medicine at Mount SinaiNew York, NY, United States; ^14^Department of Neuroscience, Icahn School of Medicine at Mount SinaiNew York, NY, United States; ^15^Friedman Brain Institute, Icahn School of Medicine at Mount SinaiNew York, NY, United States; ^16^Department of Genetics and Genomic Sciences, Icahn School of Medicine at Mount SinaiNew York, NY, United States; ^17^Mindich Child Health and Development Institute, Icahn School of Medicine at Mount SinaiNew York, NY, United States; ^18^Department of Medical Sciences, Università di TorinoTurin, Italy; ^19^Medical Genetics Unit, Azienda Ospedaliera Città della Salute e della Scienza di TorinoTurin, Italy

**Keywords:** p140Cap, postsynaptic density, synaptic transmission, synaptic plasticity, schizophrenia, autism, intellectual disability, epilepsy

## Abstract

Altered synaptic function has been associated with neurological and psychiatric conditions including intellectual disability, schizophrenia and autism spectrum disorder (ASD). Amongst the recently discovered synaptic proteins is p140Cap, an adaptor that localizes at dendritic spines and regulates their maturation and physiology. We recently showed that p140Cap knockout mice have cognitive deficits, impaired long-term potentiation (LTP) and long-term depression (LTD), and immature, filopodia-like dendritic spines. Only a few p140Cap interacting proteins have been identified in the brain and the molecular complexes and pathways underlying p140Cap synaptic function are largely unknown. Here, we isolated and characterized the p140Cap synaptic interactome by co-immunoprecipitation from crude mouse synaptosomes, followed by mass spectrometry-based proteomics. We identified 351 p140Cap interactors and found that they cluster to sub complexes mostly located in the postsynaptic density (PSD). p140Cap interactors converge on key synaptic processes, including transmission across chemical synapses, actin cytoskeleton remodeling and cell-cell junction organization. Gene co-expression data further support convergent functions: the p140Cap interactors are tightly co-expressed with each other and with p140Cap. Importantly, the p140Cap interactome and its co-expression network show strong enrichment in genes associated with schizophrenia, autism, bipolar disorder, intellectual disability and epilepsy, supporting synaptic dysfunction as a shared biological feature in brain diseases. Overall, our data provide novel insights into the molecular organization of the synapse and indicate that p140Cap acts as a hub for postsynaptic complexes relevant to psychiatric and neurological disorders.

## Introduction

Mutations in genes encoding synaptic proteins have been associated with several brain disorders, including schizophrenia, autism spectrum disorder (ASD) and developmental delay/intellectual disability (DD/ID) (De Rubeis et al., 2014; Fromer et al., 2014; Deciphering Developmental Disorders, 2015). The term “synaptopathies” has in fact been proposed to cover a broad range of clinical manifestations having synaptic dysfunction as common etiology (Grant, 2012). The synaptic genes implicated in brain diseases span several functional classes, including adhesion molecules (e.g., *NRXN1*; Kim et al., 2008), neurotransmitter receptors (e.g., *GRIN2B* ; Endele et al., 2010), voltage-gated ion channels (e.g., *SCN2A*; Rauch et al., 2012), scaffolding proteins of the postsynaptic density (PSD) (e.g., *SHANK3*; Betancur and Buxbaum, 2013) and signaling proteins (e.g., *SYNGAP1* ; Hamdan et al., 2009).

Enormous progress has been made in the characterization of the synaptic proteome (Bayes et al., 2014), and the molecular and functional dissection of specific subsynaptic complexes is likely to reveal novel genes relevant to brain pathophysiology. Amongst the recently discovered synaptic proteins is p140Cap/SNIP (Chin et al., 2000; Di Stefano et al., 2004), a scaffolding protein localized in dendritic spines (Jaworski et al., 2009) and encoded by the *SRCIN1* gene. Previous work from our laboratory and others indicates that p140Cap is key in regulating synaptogenesis, synaptic transmission and synaptic plasticity (Jaworski et al., 2009; Tomasoni et al., 2013; Repetto et al., 2014). Acute down-regulation of p140Cap in primary hippocampal neurons reduces the number of mushroom spines and proportionally increases the number of dendritic filopodia (Jaworski et al., 2009; Tomasoni et al., 2013), a defect in synaptic maturation that can also be observed in p140Cap knockout (KO) mice (Repetto et al., 2014). Notably, dendritic spine dysgenesis is a consistent neuroanatomical finding in several psychiatric disorders, including ID, ASD and schizophrenia (Penzes et al., 2011). Further, p140Cap KO mice display defective long-term potentiation (LTP) and reduced long-term depression (LTD), two forms of synaptic plasticity necessary for learning and memory. Consistently, p140Cap KO mice show impairments in object recognition, suggestive of defects in memory consolidation and retrieval cognitive defects (Repetto et al., 2014).

The molecular complexes and pathways underlying p140Cap function are largely unknown. p140Cap was originally identified as an interactor of SNAP-25 in a two-hybrid screen (Chin et al., 2000) and subsequent studies have shown that this interaction takes place in the PSD, involving also PSD95, and is required for spine morphogenesis (Tomasoni et al., 2013; Fossati et al., 2015). p140Cap had been also shown to interact with other components of the pre-synaptic compartment, such as vinexin and synaptophysin (Ito et al., 2008). Other interactions necessary for p140Cap-mediated control of spine formation and/or maturation are those with the EB3 (Jaworski et al., 2009), Endophilin A1 (Yang et al., 2015), and Src kinase (Di Stefano et al., 2007; Repetto et al., 2013, 2014). EB3 binds the plus-end of growing microtubules that can enter dendritic spines and influence their morphology (Jaworski et al., 2009). Endophilin A1 is localized both presynaptically, where it regulates synaptic vesicle endocytosis, and postsynaptically, where it controls spine morphogenesis (Yang et al., 2015). The correct functioning of both EB3 and Endophilin A1 requires p140Cap and its downstream effector cortactin, an F-actin-binding protein, that regulates the actin cytoskeleton organization (Uruno et al., 2001). The p140Cap-dependent regulation of cortactin activity might require a direct interaction as well as the suppression of the Src kinase pathway. In fact, cortactin is inhibited by Src phosphorylation (Martinez-Quiles et al., 2004), and p140Cap directly binds Src (Di Stefano et al., 2007) and suppresses its activation through binding to the C-terminal Src kinase (Csk) (Di Stefano et al., 2007; Repetto et al., 2013). Consistently, synapses isolated from p140Cap KO mice have increased phosphorylation on Src kinase Tyrosine 416 (Repetto et al., 2014) and treatment with a specific Src inhibitor ameliorates the spine defects observed in p140Cap KO neurons (Repetto et al., 2014). Beyond its role in spine morphology, p140Cap-mediated suppression of Src has been recently implicated in the homeostatic brake that restrains cocaine reward in the nucleus accumbens (Damez-Werno et al., 2016).

Given the role of p140Cap in synaptogenesis and the repercussions on cognition in its absence, we sought to capture the p140Cap molecular complexes at synapses and investigate their relevance for brain disorders. By using biochemical, proteomic, gene co-expression, and genetic data, we provide a comprehensive analysis of the p140Cap synaptic interactome. Our results reveal that p140Cap acts as a hub for synaptic proteins implicated in psychiatric and neurological disorders.

## Materials and methods

### Animals

Mixed 129Sv × C57BL/6J p140Cap heterozygous mice were generated as described in (Repetto et al., 2014). All experiments were approved and performed in accordance with the Italian law (authorization D.M. n°279/95B 27/11/1995) and dispositions of “D.L. n°116, 27/1/1992 in relation to animal use and protection in scientific research.” Male, 3-month old wild-type (WT) and p140Cap knockout (KO) littermates were used for synaptosomes preparations. Animals were sacrificed by cervical dislocation.

### Synaptosomes preparation

Synaptosomes were prepared from the telencephalon. The tissue was homogenized with a Dounce glass homogenizer and glass pestle in 8 ml ice-cold synaptosome buffer (4 mM Hepes pH = 7.3, 320 mM sucrose, 1 mM EGTA, Roche protease inhibitors 25X, 1 mM Sodium Orthovanadate, 1 mM DTT, phenylmethylsulphonyl fluoride, 1 mM sodium fluoride). The homogenate was centrifuged at 1,000 g for 10 min at 4°C. After discarding the nuclear pellet, the supernatant was centrifuged at 12,500 g for 20 min at 4°C. The pellet containing the synaptosomal fraction was resuspended in the synaptosome buffer and further centrifuged at 12,500 g for 20 min at 4°C. The final pellet (crude synaptosome fraction) was resuspended in 2 ml of ice-cold lysis buffer (150 mM NaCl, 50 mM Tris pH = 7, 5% Glycerol, 1% NP-40, 1 mM MgCl_2_, Roche protease inhibitors 25X, 1 mM Sodium Orthovanadate, 1 mM DTT, phenylmethylsulphonyl fluoride, 1 mM sodium fluoride) and immediately processed for immunoprecipitation (Figure S1).

### Western blot

Western blots were performed with Mini-PROTEAN® TGX™ Precast Gels from Bio-Rad (California 94547 USA).gradient 4–15% Gels were transferred on Nitrocellulose blotting membrane (GE Healthcare Life Sciences) using Towbin buffer (25 mM Tris, 192 mM Glycine, 20% Methanol). Membranes were blocked with Tris-buffered saline TBS (50 mM Tris p < h 7–150 mM NaCl) with 5% Milk for 1 h at room temperature, incubated with primary and secondary antibodies as indicated below, and then developed with Bio-Rad's Clarity ECL on ChemiDoc Touch Imaging System (Biorad). For western blot of crude synaptosomal proteins, 30 ug of proteins was used.

### Antibodies

Specific mouse monoclonal antibody (Mab) against p140Cap ***(clone 2A8)*** was produced at the MBC, University of Torino, as previously described (Di Stefano et al., 2007; Repetto et al., 2013). The antibodies used are as follows: Dlg4 (Mab, Abcam), Citron-N (rabbit polyclonal, Abcam), Grin2A (rMab from Millipore; rabbit polyclonal from Thermo Scientific), Grin2b (mouse monoclonal antibody, Neuromab), Grin1 rabbit polyclonal (Thermo Scientific), Shank2 (rabbit polyclonal, Synaptic System), Shank3 (rabbit polyclonal, Synaptic System), Snap25 (Mab, Synaptic System), Actin I-19 (rabbit polyclonal, Santa Cruz Biotechnology), Trio (rabbit polyclonal, Santa Cruz Biotechnology), rabbit polyclonal Camk2 (Cell Signaling), Homer-1 (rabbit polyclonal, Thermo Scientific), Cadherin-11 (Mab, Thermo Scientific), Syncam (rabbit polyclonal, Thermo Scientific), Ttc3 (rabbit polyclonal, as described in Berto et al., 2007), and Gfap (Mab, Dako-Agilent Technologies), Bassoon (rabbit polyclonal, Synaptic System). Mouse and rabbit IgGs were purchased from Santa Cruz Biotechnology. Secondary antibodies anti-mouse and anti-rabbit were purchased from Sigma Aldrich.

### p140Cap immunoprecipitation

1.25 mg of Dynabeads protein G (30 mg Dynabeads®/mL Invitrogen, Carlsbad, CA, USA) were initially washed using a Sodium Acetate buffer (100 mM Sodium acetate pH = 5, 0.1% NP-40) and incubated with 6 μg of p140Cap antibody diluted in the same buffer for 1 h at room temperature under gentle rotation. Beads were then washed twice withSodium acetate buffer and with Sodium Borate buffer (0.2 M Sodium borate pH = 9, 0.1% NP-40) Crosslinking was performed with 25 mM Dymethil Pimelimidate in 1 ml of Sodium Borate buffer for 45 min at room temperature on a wheel. Beads were washed again with Sodium Borate buffer and then with 0.2 M Ethanolamine pH = 8, 0.1% NP-40, incubated for 1 h at RT on a wheel to block the N-reactive groups and washed twice in PBS 0.1% NP-40. To remove the non-cross-linked antibody, beads were suspended in 100 mM Glycine pH = 2.5 and rapidly washed in 50 mM Tris pH = 7.4 0.1% NP-40. p140Cap-coupled Dynabeads were then washed twice with PBS 1X 0.05% Tween and then incubated with 6 mg of crude synaptosomal extracted for 2 h at 4°C. Beads were washed five times with cold lysis buffer, then resuspended in 60 μl of 2% SDS-PAGE sample buffer in reducing conditions and incubated at 70°C for 10 min. From this 1 mg was tested for Coomassie staining, 1 mg for Western blot for p140Cap to control the quality of the samples and 4 mg was used for mass spectrometry (Figure S2).

### Primary hippocampal cultures

Primary cultures were established from the hippocampus of 17.5–18.5 embryos derived from breeding of p140Cap ± mice. The dissociated cells were plated on 20 mm coverslip coated with poly-lysine at the density of 80,000 cells/coverslip and cultured in Neurobasal medium (Invitrogen) added with B27 (Invitrogen), supplemented with antibiotics, 2 mM glutamine and glutamate for 17–18 days at 37°C. Neurons were then fixed in paraformaldehyde 4% and sucrose 4% (Sigma Aldrich) for 8 min at room temperature.

### Immunofluorescence and microscopy

Immunofluorescence staining was performed using antibodies against PSD95 (1:500 mouse monoclonal Abcam) and p140Cap homemade monoclonal antibody conjugated with Alexa Fluor 647 Antibody labeling kit (Thermo Fisher) diluted 1:500. Coverslips were first incubated with anti-PSD95 and then with Alexa fluor 488 goat anti mouse (Invitrogen); after several washes of the secondary antibody, conjugated anti-p140Cap Mab was added for 1h at room temperature and then washed away. Images were acquired using a Leica SPE confocal microscope.

### Mass spectrometry

(1) *Preparation and in-gel digestion of proteins*: proteins from co-IP eluates were stacked in the top of a 4–12% NuPAGE gel (Invitrogen) and stained with R-250 Coomassie blue. Gel bands were manually excised and cut in pieces before being washed six times with 25 mM NH_4_HCO_3_ for 15 min, followed by six washes in 25 mM NH_4_HCO_3_ containing 50% (v/v) acetonitrile. Gel pieces were then dehydrated with 100% acetonitrile and incubated with 10 mM DTT in 25 mM NH_4_HCO_3_ for 45 min at 53°C and with 55 mM iodoacetamide in 25 mM NH_4_HCO_3_ for 35 min in the dark. Alkylation was stopped by adding 10 mM DTT in 25 mM NH_4_HCO_3_ (incubation for 10 min). Gel pieces were then washed again by incubation in 25 mM NH_4_HCO_3_followed by dehydration with 100% acetonitrile. Modified trypsin (Promega, sequencing grade) in 25 mM NH_4_HCO_3_ was added to the dehydrated gel pieces before overnight incubation at 37°C. Peptides were extracted from gel pieces in three sequential extraction steps (each 15 min) using 30 μl of 50% acetonitrile, 30 μl of 5% formic acid, and finally 30 μl of 100% acetonitrile. The pooled supernatants were dried under vacuum. (2) *Nano-LC-MS/MS analyses*: the dried extracted peptides were resuspended in 5% acetonitrile and 0.1% trifluoroacetic acid and analyzed by online nanoLC-MS/MS (UltiMate 3000 RSLCnano and Q-Exactive Plus, Thermo Scientific) with 2 replicates per sample. Peptides were sampled on a 300 μm x 5 mm PepMap C18 precolumn and separated on a 75 μm × 250 mm C18 column (PepMap, Dionex). The nanoLC method consisted of a 120-min gradient at a flow rate of 300 nl/min, ranging from 5 to 37% acetronitrile in 0.1% formic acid for 114 min, before reaching 72% acetronitrile in 0.1% formic acid for the last 6 min. Spray voltage was set at 1.6 kV; heated capillary was adjusted to 270°C. Survey full-scan MS spectra (m/z = 400–1,600) were acquired with a resolution of 70,000 after accumulation of 10^6^ ions (maximum filling time 200 ms). The 10 most intense ions were fragmented by higher-energy collisional dissociation after accumulation of 10^5^ ions (maximum filling time 50 ms). MS and MS/MS data were acquired using the software Xcalibur (Thermo Scientific). (3) *Data analyses*: RAW files were processed using MaxQuant, version 1.5.1.2 (Cox and Mann, 2008). Spectra were searched against the Uniprot database (*Mus musculus* taxonomy, March 2015 version) and the frequently observed contaminants database embedded in MaxQuant. The I = L option was activated. Trypsin was chosen as the enzyme and 2 missed cleavages were allowed. Peptide modifications allowed during the search were: carbamidomethylation (C, fixed), acetyl (Protein N-ter, variable) and oxidation (M, variable). Minimum number of unique peptides was set to 1. The matching between runs option was activated. The mass spectrometry proteomics data have been deposited to the ProteomeXchange Consortium via the PRIDEpartner repository (Vizcaino et al., 2016) with the dataset identifier PXD004215. (4) *Statistical analyses*: The following steps were mainly performed using the Perseus toolbox (version 1.5.1.6) available in the MaxQuant environment. Proteins identified in the reverse and contaminant databases, identified with less than 2 razor + unique peptides, or exhibiting less than 6 iBAQ values in one condition (3 biological replicates with 2 analytical replicates each for control and p140Cap co-IPs) were discarded from the list. iBAQ values of the 1952 remaining proteins. After log2 transformation, iBAQ values were normalized by condition-wise centering, missing data imputation was realized (replacing missing values by a constant weak value calculated independently for each injected sample as the 2.5-percentile value of the column) and statistical testing of differential abundances between control and p140Cap conditions were conducted using welch *t*-testing. To consider a protein as a potential binding partner of p140Cap, it must has passed our significance criteria: FDR threshold of 1% on *p*-values using the Benjamini-Hochberg method and a minimum 4-times enrichment in p140Cap WT samples compared to the KO controls.

### Bioinformatic analyses of MS data

Throughout the study, over-representation of annotation terms (disease, function etc.) was estimated by use of the hypergeometric distribution to test whether the number of selected proteins is larger than would be expected by chance;

$$\begin{array}{l} p=\;1-\sum_{i=0}^{k-1}\frac{\left(\frac{M}{i}\right)\left(\frac{N-M}{n-i}\right)}{\left(\frac{N}{n}\right)} \end{array}$$

where *N* is a total number of proteins in the background distribution, *M* is the number of genes within distribution that are annotated to the node of interest, *n* is the size of the list of genes of interest and *k* is the number of genes within the list, which are annotated to the node. Obtained *p*-values were adjusted for multiple testing by Bonferroni correction at 0.05 or 0.01 significance levels as indicated.

For literature comparison, a list of 6,688 proteins was selected as a synaptic *universe* based on combined results from 35 published synapse proteome studies (in preparation; Supplementary Table S2). Protein lists from those studies were curated, mapped to stable IDs (Entrez, Uniprot, MGI) for human and mouse and combined into single list containing only unique protein/gene name, IDs and publication source (PMID). Summary data for the 35 publications with associated metadata (PMID, year, method, species and number of proteins found) are presented in Supplementary Table S2).

Enrichment analysis of annotation in the interactome was performed in R, specifically using the Bioconductor package ClusterProfiler for Gene Ontology (GO) and KEGG enrichment analysis (Yu et al., 2012) and Bioconductor ReactomePA package for pathway over-representation analysis (http://bioconductor.org/packages/release/bioc/html/ReactomePA.html). For each enrichment type (GO, KEGG, Reactome), two background sets of proteins were used: (1) the default mouse genome list from Bioconductor and (2) our list of 6688 published synaptic proteins-to reveal the consistency in enriched terms. *p*-values, adjusted for multiple comparison p.adjust and *q*-values for false discovery rates (FDR) are provided in Supplementary Table S3.

For disease enrichment the annotation data were standardized using MetaMap (Aronson and Lang, 2010) and NCBO Annotator (Whetzel et al., 2011; Musen et al., 2012) to recognize terms found in the Human Disease Ontology (HDO) (Schriml et al., 2012) Recognized enriched disease ontology terms were then associated with gene identifiers and stored locally. Disease term enrichment', for the p140Cap dataset', could then be calculated using the Topology-based Elimination Fisher method (Alexa et al., 2006) found in the topGO package (http://topgo.bioinf-mpi-inf.mpg.de/), together with the standardized OMIM and Ensembl variation gene-disease annotation data (14111 gene-disease associations), mapped onto the full HDO tree.

A PSD network was constructed from a list of 1,443 proteins obtained from a study of the PSD in the human brain (Bayes et al., 2011). Protein-protein interactions were obtained by mining publicly available databases: HIPPIE (Schaefer et al., 2012), BioGRID (Chatr-Aryamontri et al., 2015), IntAct (Kerrien et al., 2012), and performing an InterologWalk over different species using Bio::Homology::InterologWalk (Gallone et al., 2011). The connected PSD network consists of 1,312 proteins and 8,031 protein interactions (i.e., 131 proteins could not be connected into the network). This PSD network was clustered making use of the spectral properties of the network; the network being expressed in terms of its eigenvectors and eigenvalues, and partitioned recursively (using a fine-tuning step) into communities based on maximizing the Modularity clustering measure (Newman, 2006; Simpson et al., 2010). Where first we found the node which, when moved from one community to the other, gave the maximum change in Modularity. This node's community was then fixed and we repeat the process until all nodes had been moved. The whole process was repeated from this new state until the change in the Modularity, between the new and old state, was less than the predefined tolerance. The modularity of the full PSD network was found to be 0.36.

Robustness of the full PSD network communities was assessed by running the algorithm 500 times, randomly selecting 80% of the network node set and related interactions each time. The package clusterCons (Simpson et al., 2010) was used to build a consensus matrix from which to test the robustness of the communities, and proteins found inside the communities. Community and protein robustness values range from 0, indicating no confidence in existing through to 1, indicating absolute confidence in the cluster existing. Community robustness values range from 0.1 to 0.7, and from 0.01 to 0.9 for protein robustness. Clusters C7, C17, C22, and C23 showed evidence of being robust, with community robustness values 0.2, 0.4, 0.3, and 0.3 respectively.

We also tested the significance of annotation enrichment in each cluster using the Hypergeometric distribution formula. *P* ≤ 10^−2^, were tested for their strength of significance by recording the percentage of *P*-values found from every community/annotation combination, lower than or equal to the observed *P*-value, when 1,000 random permutations of the annotation labels were made. *P*-values found with a strength of significance < 1% were considered statistically significant. *P*-values values were also tested against a more stringent Bonferroni correction at the 0.05 (^*^), 0.01 (^**^), and 0.001 (^***^) significant levels, and highlighted throughout the enrichment tables.

### Gene co-expression analysis

Gene co-expression was evaluated on 18 human-mouse conserved co-expression networks (CCNs) (Piro et al., 2011), where a link of co-expression reflects the human and mouse independent gene co-expression in the corresponding single-species networks. Statistical significance of p140Cap interactors co-expressed with p140Cap was assessed by hypergeometric distribution. Statistical significance of the co-expression between p140Cap interactors was assessed by evaluating the *p*-value of the z-score corresponding to the number of links found in the single CCNs, based on 100 randomization of the same CCNs.

## Results

### Quantitative proteomic analysis of p140Cap synaptic interactome

**Figure 1:**
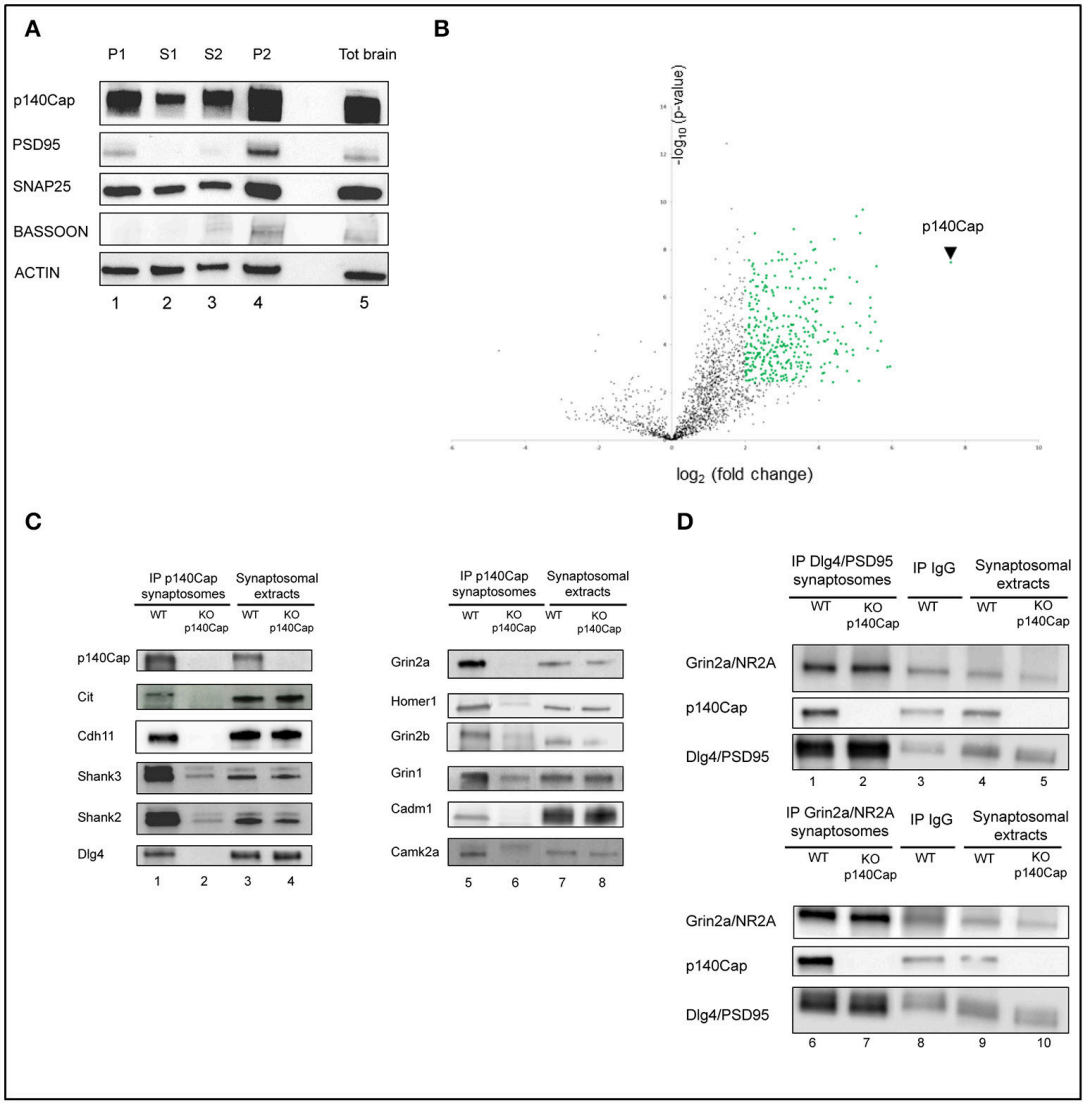
Identification and validation of p140Cap interactors. Enrichment of synaptic markers in the synaptosomal preparation. **(A)** Validation of the synaptosomes. Western blot for p140Cap, PSD-95, Snap25, ***Bassoon*** and beta-actin fractions isolated during synaptosomal preparation. Lane 1, nuclear fraction (P1); lane 2, first cytosolic fraction (S1); lane 3, second cytosolic fraction (S2); lane 4, crude synaptosome (P2), lane 5, total brain extracts (see Figure S1 for details). **(B)** Statistically enriched proteins in the p140Cap IP. The Volcano plot represents the log_10_(*p*-value, y axis) plotted against the log_2_(fold change, x axis) for proteins quantified in p140Cap IPs from WT and p140Cap KO, used as negative control. 357 different proteins, including p140Cap (arrowhead) were found significantly enriched in WT samples (FDR ≤ 1%, ≥ 4-fold enrichment) are shown as green dots **(C)** Validation of synaptic p140Cap interacting proteins identified in the interactome. Lane 1 and 5, p140Cap IP in WT animals; lane 2 and 6, p140Cap IP in p140Cap KO animals; lane 3 and 7, input from WT synaptosomes; lane 4 and 8, input from KO synaptosomes. Co-immunoprecipitated proteins are shown on the left, along with their rank in MS. **(D)** Reverse validation for p140Cap interacting proteins Dlg4/PSD95 and Grin2a/NR2A. Lane 1, Dlg4/PSD95 IP in WT animals; lane 2, Dlg4/PSD95 IP in p140Cap KO animals; lane 4, input from WT synaptosomes; lane 5, input from KO synaptosomes; lane 6, Grin2a/NR2A IP in WT animals; lane 7, Grin2a/NR2A IP in p140Cap KO animals; lane 9, input from WT synaptosomes; lane 10, input from KO synaptosomes. Lane 3 and lane 8 are control IP with IgGs in WT animals.

To identify the p140Cap protein complexes and the related functional pathways at synapses, we performed a quantitative proteomic analysis of p140Cap immunoprecipitates from mouse crude synaptosomes preparations, using p140Cap KO mice (Repetto et al., 2014) as negative control (Figure S1). The quality of the preparations was verified by testing the enrichment of synaptic proteins in the crude synaptosome fraction compared to total brain extracts. PSD-95, SNAP-25, and Bassoon, as well as p140Cap, were enriched in synaptosomes (Figure 1A).

Three distinct crude synaptosomes were prepared from 3 month-old WT and p140Cap KO male mice and synaptosomal extracts were immunoprecipitated using p140Cap monoclonal antibody. The specificity of the immunoprecipitates (IP) was confirmed by Coomassie staining and western blot (Figure S2). In the Coomassie-stained gel, we detected a discrete band of ~150 kDa, corresponding to the expected molecular weight for p140Cap in SDS-PAGE in the IPs from WT samples but not from p140Cap KO synaptosomes (Figure S2A). Similarly, a p140Cap immunoreactive band was observed in the p140Cap IPs from WT but not from KO synaptosomes (Figure S2B). The Coomassie-stained gel reveals that several proteins seem to selectively co-immunoprecipitate with p140Cap from WT samples as compared with the KO ones. Overall, these data indicate that the p140Cap immunoprecipitation is highly specific, thus making these samples suitable for the identification of p140Cap interactors by mass spectrometry (MS).

To identify p140Cap-binding partners, we applied label-free quantitative MS-based proteomics to the p140Cap IPs from WT and KO synaptosomes. Proteins eluted from the IPs were in-gel digested and the resulting peptides analyzed by nanoliquid chromatography coupled to tandem MS (two analytical replicates per sample). Identities and intensities of the recovered peptides and proteins in each sample were obtained using MaxQuant (Cox and Mann, 2008). After filtering and statistical analysis of iBAQ values (Schwanhausser et al., 2011), 352 (351 interactors plus p140Cap) proteins enriched in the WT samples were identified (Supplementary Table S1), as represented in the Volcano plot (Figure 1B).

To validate the proteomic findings, we selected eleven candidates whose alterations might underlie the dendritic spine defects, the impairments in LTP/LTD, and the learning and memory deficits we previously observed in the p140Cap KO mice (Repetto et al., 2014). We then tested the presence of the selected proteins on the p140Cap IPs. We tested and validated p140Cap interactions with Citron-N, already known to interact with p140Cap (Repetto et al., 2014); the NMDA receptor subunits Grin2a, Grin2b and Grin1, the scaffold proteins Dlg4/PSD95, Shank2, Shank3, Homer1, and Camk2a. Alterations in expression or localization of several of these interactors are known to affect LTP/LTD and cognitive functions (Grant, 2012; Luscher and Malenka, 2012; Robison, 2014; Sweatt, 2016). Further, we validated the interaction with the adhesion molecules Cadherin-11 (Cdh11) (Manabe et al., 2000) and Cadm1/Syncam (Frei and Stoeckli, 2016; Figure 1C). We also verified that three proteins (Ttc3, Trio, and Gfap), placed very low in the interactome, were only non specifically immunoprecipitated with anti p140Cap Mab (Figure S3). Further, we performed a reverse validation: we immunoprecipitated PSD95 and Grin2a and verified the presence of p140Cap (Figure 1D). The validation of the proteomic data across a range of various enrichment levels above the fixed cut-off indicates that the p140Cap synaptic interactome we isolated contains bona fine p140Cap-containing macromolecular complexes.

### Synaptic localisation of p140CAP complexes with comparative study of reported synaptic datasets

To further map the p140Cap interactome, we searched published neuronal proteome datasets for p140Cap and its interactors. We found that p140Cap was reported in the majority of the published PSD datasets (Supplementary Table S2). In a few co-IP studies, p140Cap was detected in complexes with PSD95 (Dosemeci et al., 2007), mGluR5 (Farr et al., 2004), Chmpb2 (Chassefeyre et al., 2018), and Snap25 (Fossati et al., 2015). Further, we searched for the 351 identified interactors of p140Cap across the published PSD proteomic datasets and found an exceptionally high enrichment (order of E-14 of significance in average) (Supplementary Table S2).

We also sought to verify whether p140Cap and associated proteins were found in presynaptic preparations as well, and we found that p140Cap was detected in the presynaptic compartment in several studies (Gronborg et al., 2010; Boyken et al., 2013; Weingarten et al., 2014). Specifically, it was detected in complexes with Rims1, Syn1-2-3, Git1, and Wasf1 in co-IP experiments on presynaptic protein complexes (August B Smit, personal communication). However, it was not reported in other studies focusing on synaptic vesicles (SV) or SV-recycling proteins (Morciano et al., 2005, 2009; Gorini et al., 2010; Brinkmalm et al., 2014; Wilhelm et al., 2014). p140Cap–interacting proteins, in turn, were found enriched in presynaptic datasets, although with a lower score than that found in postsynaptic studies (Supplementary Table S2). Therefore, our proteomic data, in agreement with previous studies, place p140Cap mainly, but not exclusively, in the PSD.

**Figure 2:**
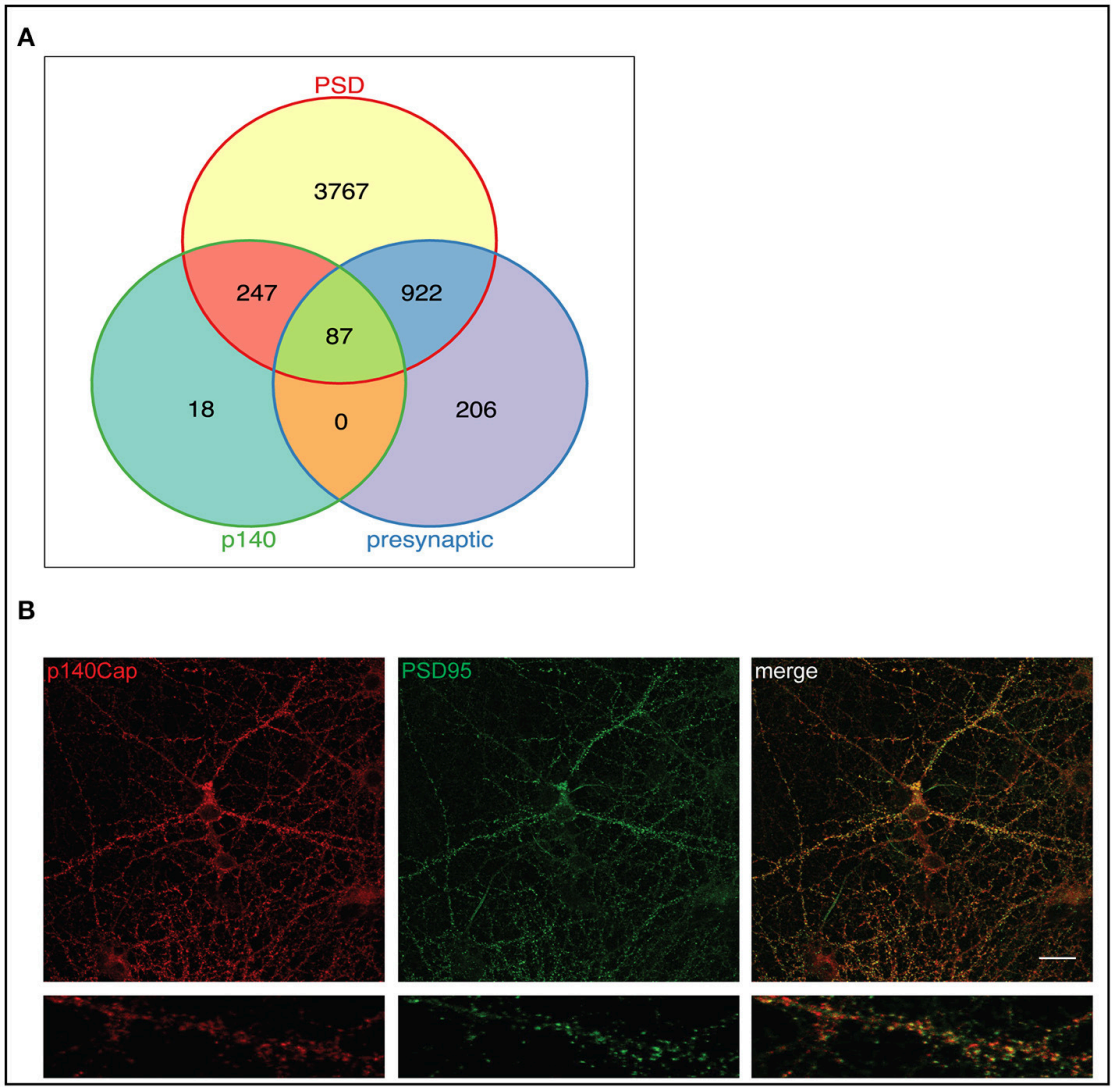
p140Cap interacting proteins distribution in pre- and postsynaptic compartments and functional modules of the p140Cap interactome. **(A)** Venn diagram showing the overlap of 352 p140Cap interactors (green) mapped in pre- (purple) and postsynaptic (yellow) datasets. **(B)** p140Cap co-localization with PSD95 on primary hippocampal neurons. DIV 17 hippocampal neurons were stained with antibodies to p140Cap (red) and to PSD95 (green). Merge is shown on the right (Upper panels) Scale bar 20 μm. Magnification of a segment of dendrite is shown in lower panels.

We then examined all the p140-interacting proteins to evaluate their distribution between pre- and postsynaptic complexes. The two lists showed a high degree of overlap, which was expected since the majority of synaptic proteins are found in both compartments. Figure 2A shows the distribution of the p140-interacting proteins between PSD (5023 proteins from 25 postsynaptic studies and presynaptic compartment (1215 proteins from 9 presynaptic studies). 334 of the 352 p140Cap interactors overlap with these datasets. Of these proteins, none were described as specifically presynaptic, whereas the majority was described as specifically postsynaptic (247) or found in both compartments (87). This further supports a predominantly postsynaptic localization of the p140Cap complexes.

The presence of p140Cap in the PSD has been previously shown in rat synaptic fractionation, where it is enriched in the PSD fraction together with PSD95 (Mclean Colin et al., 2016). To further validate the presence of p140Cap in the PSD, we performed a localization experiment on primary mouse hippocampal cultures. Staining of both p140Cap and PSD95 in Figure 2B shows a co-localization of these two proteins at synapses. All together, these results strongly indicate that the p140Cap-associated proteins share common cellular functions and fall within specific substructures of the PSD.

### Functional enrichment analyses of the p140CAP-containing protein complexes

To obtain a functional perspective of the p140Cap interactome, we tested its enrichment against KEGG, GO ontology and Reactome databases (Supplementary Table S3). We ran analysis against two reference (background) lists: (1) the default mouse genome list provided by Bioconductor and (2) a combined list of 6688 proteins from published synapse proteomics studies, to reveal more specific p140Cap associated terms compared to the whole synaptosome. Both reference lists demonstrated a very high level of consistency in top enriched terms. However, using the synaptic as background gives more conservative (i.e., lower) significance values for enriched terms. The full lists for enrichment results are presented in Supplementary Table 3.

For the enriched terms listed here, *p*-values were corrected for multiple testing as described in the methods. Based on Molecular Function (MF) ontology, the 351 p140Cap-interacting proteins were significantly enriched for the “actin binding” term (*P* = 2.79E-17), which is in line with the previously described functions of p140Cap (Repetto et al., 2014). Additionally, we found that “glutamate receptor binding” (*P* = 3.46E-11), “PDZ domain binding” (*P* = 1.22E-06) and “GTPase regulator activity” (*P* = 2.86E-04) terms were also significantly increased (Supplementary Table S3). Among the top Cellular Compartment (CC) terms were “postsynaptic membrane” (*P* = 3.08E-27), “actin cytoskeleton” (*P* = 2.87E-14), and “neurotransmitter receptor complex” (*P* = 8.53E-10). This is in agreement with Biological Process (BP) GO results, where the top enriched terms were “chemical synaptic transmission” (*P* = 1.81E-15), “actin cytoskeleton organization” (*P* = 4.37E-11), “regulation of synaptic plasticity” (*P* = 1.96E-09) and “regulation of protein complex assembly” (*P* = 3.63E-06) (Supplementary Table S3). Analysis of enrichment against the Reactome pathway database revealed the overrepresentation for “Transmission across Chemical Synapses” (*P* = 7.05E-15), “Unblocking of NMDA receptor, glutamate binding and activation” (*P* = 4.90E-10), “Cell-cell junction organization” (*P* = 1.44E-08), “Ras activation upon Ca influx through NMDA receptor” (*P* = 2.49E-08), “L1CAM interactions” (*P* = 1.00E-07) (Supplementary Table S3).

**Figure 3:**
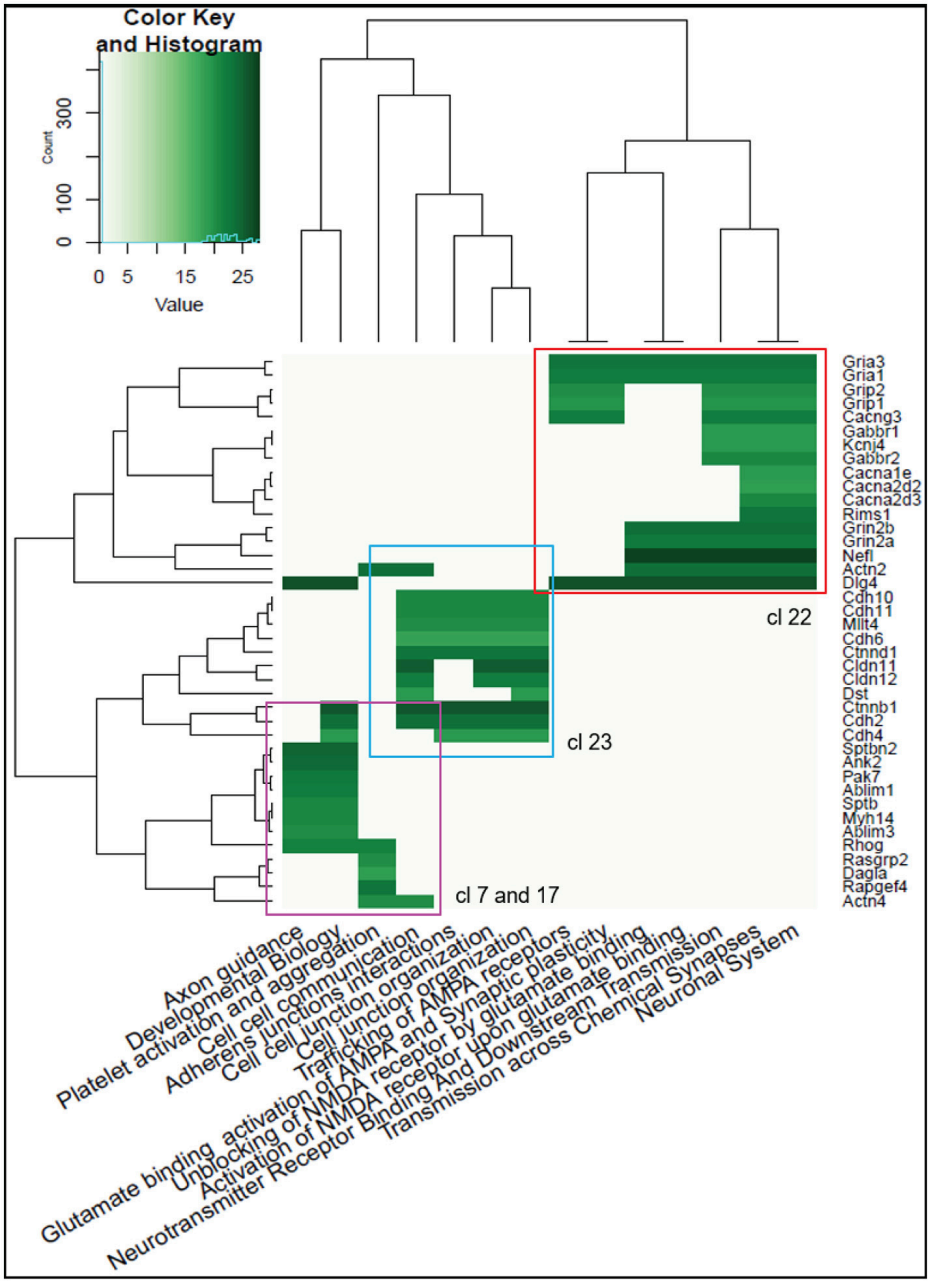
Heatmap of the Reactome Pathway enrichment analysis clustering. Intensity is based on the average relative protein abundance in MS. Three main modules are observed: upper right, Module 1; center, Module 2; and, bottom left, Module 3.

To classify proteins into functionally related modules, we checked for the correlation between the experimentally measured protein abundances and Reactome pathway terms for the top 174 interacting proteins with the highest significance. Three “modules” could be detected (Figure 3). Module 1 (Figure 3, upper right) is related to “Unblocking and activating NMDA receptors,” “Trafficking of AMPA receptors” and “Chemical signaling through the synapse” Reactome terms (including Gria1, Gria3, Grin2B, and Dlg4). Module 2 (Figure 3, center), is largely composed of cadherin-, claudin- and catenin-related proteins involved in “Cellular adhesion and junction” functions. Module 3 (Figure 3, bottom left) includes proteins associated in “Axon Guidance” and “Developmental Biology” function.

Taken together, the functional enrichment analysis from these three distinct sources (GO, Reactome and KEGG) indicate that the p140Cap interactors exhibit functions relevant to synaptic structure, synaptic transmission and plasticity.

### The p140CAP interactome within the structure of the PSD

**Figure 4:**
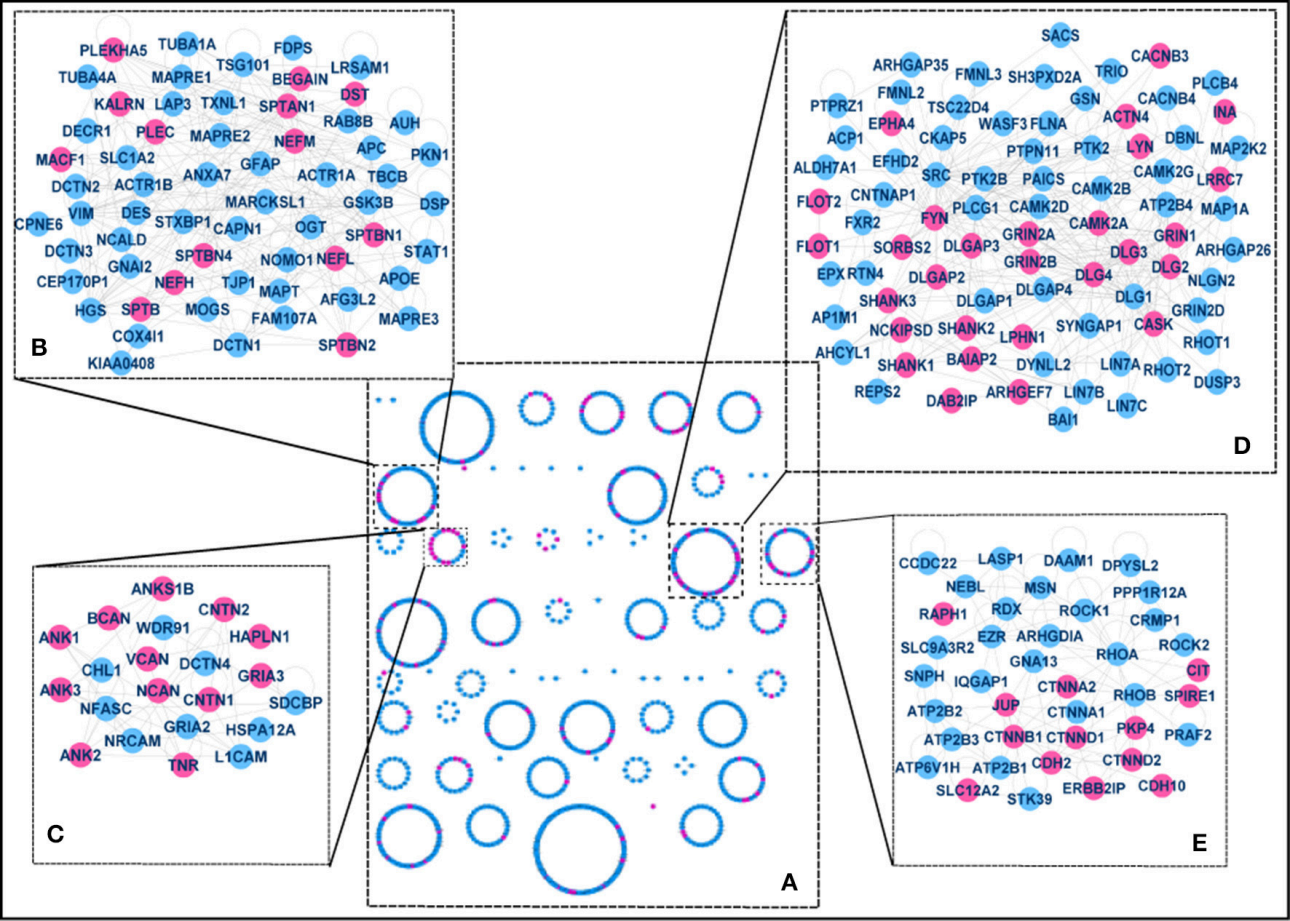
The p140Cap-interactome within the PSD **(A)** Clusters in the full PSD network. 60 structural clusters where obtained, including 4 significantly enriched for p140Cap–interacting proteins (purple), magnified in panels B–E. **(B)** Components of Cluster 7. The cluster is overrepresented with “cytoskeleton organization,” “actin cytoskeleton organization,” and “cell junction assembly” terms and contains p140Cap interactors from Module 3. **(C)** Components of Cluster 17. This cluster is associated with trans-synaptic signaling and contains p140Cap interacting proteins from Module 3. **(D)** Components of Cluster 22. The cluster includes the main MAGUK and scaffold proteins and associated with “glutamate receptor signaling pathway,” “endocytosis,” “cell-cell signaling,” and “synaptic transmission” terms and contains the majority of the proteins from Module 1. **(E)** Components of Cluster 23. The cluster is associated with cell adhesion function and cell junction functions and is enriched with proteins from Module 2.

Given the enrichment in PSD (Figure 2A), we examined previously described PSD protein network models to assess whether the p140Cap-interacting proteins form structural composites within the PSD or are rather randomly distributed throughout the network. We built a protein-protein interaction (PPI) network based on a human PSD protein dataset combined with the interaction data from several public databases (see Materials and Methods and Mclean Colin et al., 2016 for details). Spectral cluster analysis was performed on the network as described in Mclean Colin et al. (2016). Within the PSD network of 1,312 proteins, we identified 60 structural clusters based on the network topography. Essentially each cluster contains proteins that are more densely connected to other proteins within the cluster than to proteins elsewhere in the network (Figure 4).

Functional enrichment was tested for each of the clusters to assess structure/function relationships within the molecular network (the distribution of the proteins between the clusters and enrichment results are shown in Supplementary Table S4). We then mapped the p140Cap-interacting proteins onto the network and identified the clusters that are more strongly enriched for p140Cap-interacting proteins compared to a random distribution. We found 4 clusters with significant over-representation for p140Cap-interacting proteins: Cluster 7 (Figure 4B) (14/61, *P* = 3.5E-02), Cluster 17 (12/21, *P* = 3.62002E-06), Cluster 22 (28/80, *P* = 7.05E-07), and Cluster 23 (13/39, *P* = 1.4E-03). Cluster 22 (Figure 4D) is overrepresented with GO BP “glutamate receptor signaling pathway,” “endocytosis,” “cell-cell signaling,” and “synaptic transmission” terms and contains the main PSD scaffolding proteins of the MAGUK family and their interactors. This cluster was also significantly enriched with proteins from functional Module 1 (see Figure 3). Cluster 23 (Figure 4E), was found to be associated with GO BP term “cell junction assembly,” and was enriched with proteins from Module 2 (see Figure 3). Cluster 7 (Figure 4B), was overrepresented with the “cytoskeleton organization,” “actin cytoskeleton organization” and “cell junction assembly” terms and it contains proteins from Module 3 (Figure 3, Supplementary Table S4).

### Co-expression analysis of p140CAP interactors

To assess whether the expression of p140Cap interacting proteins could also be co-regulated, we isolated the top significant 174 p140Cap-interacting proteins with an unequivocal Entrez gene ID and tested their co-expression using 18 human-mouse conserved co-expression networks (CCNs) (Piro et al., 2011).

We found a statistically significant number of p140Cap interactors co-expressed with p140Cap in “All Samples,” “Normal Tissues,” “Brain,” and “Central Nervous System” conserved co-expression networks (Supplementary Tables S5, S6). The co-expression across p140Cap interactors was also significant in six CCNs (Supplementary Table S7), including “All Samples,” “Normal Tissues,” “Brain,” and “Central Nervous System” networks (Supplementary Table S8).

**Figure 5:**
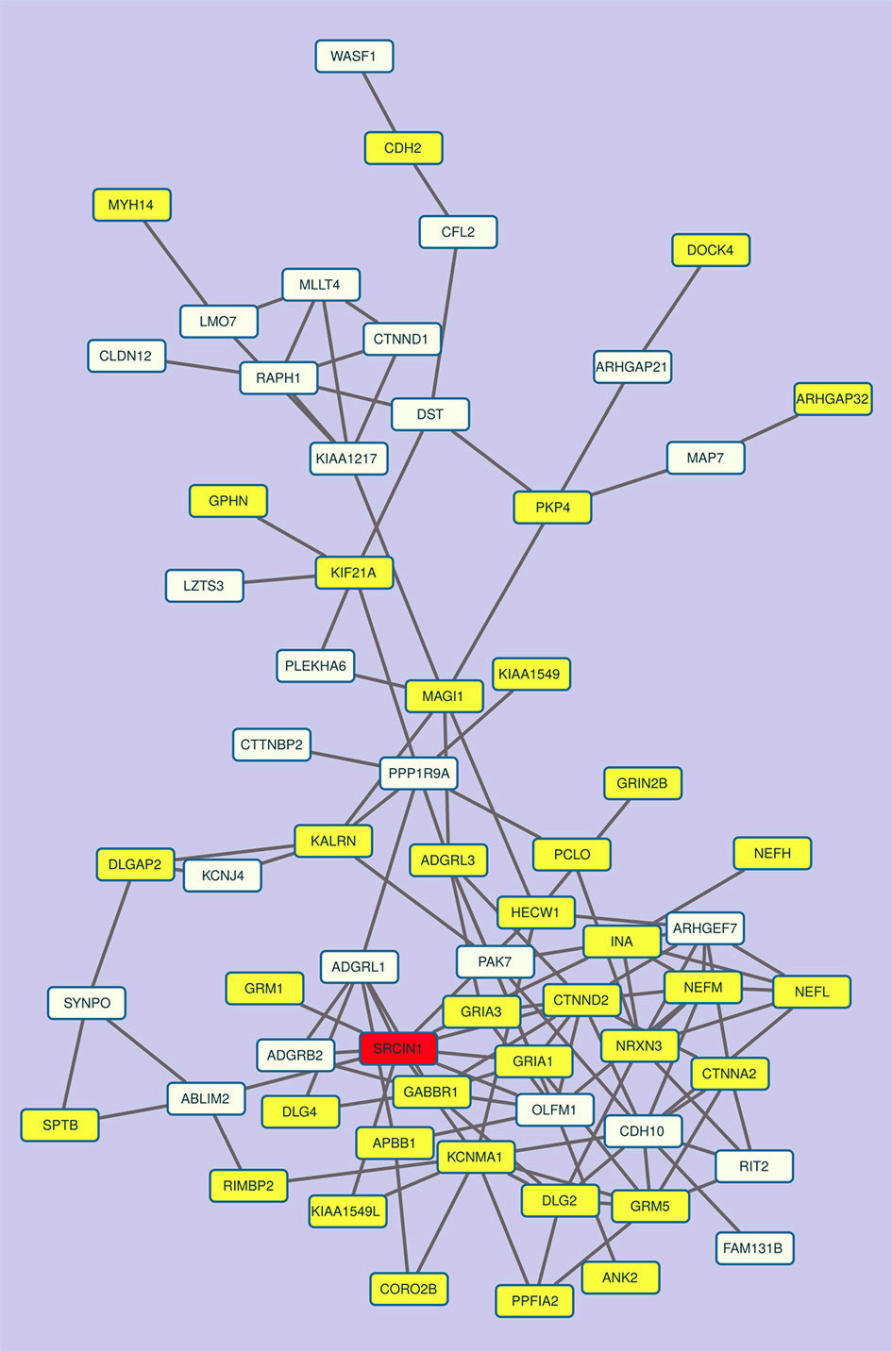
The co-expression sub-network of p140Cap and its interactors contains relevant disease genes. The most connected component of the co-expression network in “All Tissues CCN.” p140Cap is shown in red, disease genes are in yellow.

Overall, these data show that the network of p140Cap-interacting proteins also reflects a gene co-expression cluster (Figure 5), indicating that the components of the p140Cap interactome might undergo coordinated mechanisms of tissue-specific transcription control.

### Disease enrichment of p140Cap related proteins

Synaptic dysfunction has emerged as a common theme in neurological and psychiatric disorders, and recent large-scale genetic studies in ASD and schizophrenia (De Rubeis et al., 2014; Fromer et al., 2014) have further strengthened this hypothesis. The pathophysiological mechanisms might include abnormal expression of discrete synaptic proteins or the inability to organize into functional macromolecular complexes (Bayes et al., 2011). We investigated the disease enrichment of the genes encoding p140Cap interacting molecules (351 genes), using gene-disease annotation data collected from OMIM (http://omim.org/) and Ensembl variation (Chen et al., 2010) databases mapped onto the HDO tree using the topOnto package (https://github.com/statbio/topOnto). These analyses yielded a core background list of 14,111 gene-disease associations mapped onto 1,491 HDO terms. We also considered lists of risk genes and loci emerged from recent studies, including ASD (Sanders et al., 2015), epilepsy (EuroEPINOMIC-RES Consortium, 2014), Bipolar Disorder (Kataoka et al., 2016) and Schizophrenia (Fromer et al., 2014; Schizophrenia Working Group of the Psychiatric Genomics, 2014). The 826 gene-disease associations resulting from these analyses were then merged with the annotation set from the databases, yielding a total of 14,357 gene-disease associations mapped onto 1,491 HDO terms. Using the Topology-based Elimination Fisher method (Alexa et al., 2006), we found that the p140Cap dataset is significantly enriched for genes associated with schizophrenia (*P* = 2.1E-9), ASD (*P* = 7.9E-6), bipolar disorder (*P* = 5.3E-5), ID (1.1E-3), and epilepsy (1.7E-3) (Table 1, Supplementary Table S9).

**Table 1:** Disease enrichment of p140Cap related proteins.

**TERM.ID**	**Term**	**Annotated**	**Significant**	**Expected**	**classicFisher**	**elimFisher**
DOID:5419	Schizophrenia	1600	65	30.54	2.1E-09	2.1E-09
DOID:12849	ASD	338	20	6.45	7.9E-06	7.9E-06
DOID:3312	Bipolar disorder	981	37	18.72	5.3E-05	5.3E-05
DOID:1059	Intellectual disability	490	20	9.35	1.18E-03	1.18E-03
DOID:1826	Epilepsy	331	15	6.32	1.78E-03	1.78E-03

*The table shows the 5 top terms for the TopGo disease enrichment with results from Fisher and elimFisher tests. The agreement among the orders of magnitude for both methods indicates the strong over-representation of respective disease genes among the p140Cap interacting proteins*.

Moreover, a further and independent evidence in supporting the role of p140Cap in neurological diseases came from the observation that the largest connected component of the sub-network of co-expression links of p140Cap-interactors among themselves and p140Cap in the All Tissues CCN contains several genes labeled as Disease Genes and involved in neurological diseases. These results indicate that p140Cap pathways, defined by both protein-protein interactions and gene co-expression data, are relevant to psychiatric and neurological disorders.

## Discussion

The structure and the combinatorial use of a handful of modulatory elements give rise to complexity at subcellular, cellular, and systems level at the synapse (Grant et al., 2016). The loss or modification of key synaptic proteins can directly affect such network, ultimately impacting synaptic function. Our results allow to propose that the p140Cap adaptor molecule acts as a key hub of synaptic networks. Our work is the first comprehensive proteomic analysis of the synaptic interactome of p140Cap, which is indispensable for dendritic spine initiation and maturation and forms of hippocampal synaptic plasticity (Repetto et al., 2014). Our data show that the most prominent localization of p140Cap is within the PSD, with a significant enrichment for pathways related to receptors trafficking and chemical signaling through the synapse, cellular adhesion and junction, and axon guidance. We also provide evidence that the p140Cap interactome forms a gene co-expression module. Although p140Cap itself has not been implicated in brain disease yet, our results unveil a robust association of the p140Cap interactome with neurological and psychiatric disorders, including schizophrenia, ID, epilepsy and ASD.

Some of the p140Cap-interacting proteins associated to brain disorders have unknown functions in the brain. By mapping them to the synaptic p140Cap interactome, our study provides functional cues on these disease genes, including *ROGDI, FRMD4A*, and *TRANK1*. Loss of *ROGDI*, encoding a protein with unknown function, causes Kohlschutter-Tonz syndrome (MIM 614574), which presents with psychomotor delay, early-onset intractable seizures, variable ID correlating with the severity of seizures, and amelogenesis imperfect (Schossig et al., 2012). Genetic disruption of *FRMD4A* causes a syndrome characterized by congenital microcephaly accompanied by agenesis of corpus callosum and/or partial hypoplasia of the vermis and cerebellum and ID (Fine et al., 2015) (MIM 616819). *FRMD4A* regulates cell polarity in non-neuronal cells (Ikenouchi and Umeda, 2010) and the microcephaly and brain malformations reported in carriers of *FRMD4A* mutations are compatible with defects in cell polarity in neuronal progenitors and/or newborn neurons. Beyond early brain development, our data also point to a synaptic function for *FRMD4A*. *TRANK1* is a GWAS-associated locus for both schizophrenia (Schizophrenia Working Group of the Psychiatric Genomics, 2014) and bipolar disorder (Chen et al., 2013; Muhleisen et al., 2014), but its functional roles in neurons are obscure. Again, our results locate it in the p140Cap synaptic network.

There is robust evidence supporting the hypothesis that synaptic dysfunction is a shared mechanism underlying a broad range of brain disorders, and our data further corroborate it. For example, alterations in glutamatergic transmission can result in a broad spectrum of psychiatric conditions. In our dataset, we detected AMPA and NMDA receptor subunits associated with schizophrenia (i.e., *GRIA1* and *GRIN2A*) and with a spectrum of DD/ID, ASD and seizures, often in co-morbidity (*GRIA3, GRIN1, GRIN2A*, and *GRIN2B*). Glutamatergic transmission is intertwined with synaptic calcium dynamics (Higley and Sabatini, 2012), and large-scale genetic studies have shown that disruption of voltage-gated calcium channels is a pathological mechanism across psychiatric disorders (Cross-Disorder Group of the Psychiatric Genomics, 2013). Notably, this pathway holds potential for rapid therapeutic intervention (Lencz and Malhotra, 2015). Seven VGCC subunits were found in the p140Cap interactome and four of them are associated to disease. *CACNA1A* haploinsufficiency can cause episodic ataxia type 2 (MIM 108500), familial hemiplegic migraine type 1 (MIM 141500) and spinocerebellar ataxia 6 (MIM 183086). More recently, *CACNA1A* mutations have been described in individuals diagnosed with ID, epilepsy, ADHD and/or ASD (Damaj et al., 2015). *CACNA2D1* and *CACNA2D2* are both associated with epilepsy and ID (Edvardson et al., 2013; Pippucci et al., 2013; Vergult et al., 2015), while *CACN2D3* has been recently implicated in ASD (De Rubeis et al., 2014). Interestingly, none of the reproducible GWAS-associated loci in schizophrenia *CACNA1C, CACNA1H*, and *CACNB2* (Schizophrenia Working Group of the Psychiatric Genomics, 2014) was detected in our dataset. However, several RIM proteins that are necessary for VGCCs regulation, including the schizophrenia-associated *RIMS1* (Schizophrenia Working Group of the Psychiatric Genomics, 2014), are detected in the p140Cap interactome.

Psychiatric manifestations are often accompanied by defects in neuronal morphogenesis, and abnormalities of dendritic spines (either the number or the shape), which are one of the most robust neuroanatomical correlate of ID and often a finding in ASD and schizophrenia (Penzes et al., 2011). p140Cap and many of its interactors play a role in the formation, maturation and maintenance of dendritic spines. Specifically, deficiency of p140Cap results in an excess of immature filopodial-like protrusions at the expenses of mature mushroom spines (Tomasoni et al., 2013; Repetto et al., 2014). Depletion, ablation or mutations of many p140Cap interactors, including *CNKSR2* (CNK2) (Lim et al., 2014), *CTNNB1* (β-catenin) (Okuda et al., 2007), *CTNND2* (δ-catenin) (Arikkath et al., 2009), phenocopy these defects. Conversely, other p140Cap-associated proteins are needed for spine maintenance and their silencing results in reduced spine density and/or spine shrinkage, as documented for CASK (Chao et al., 2008) and ANK3 (Smith et al., 2014). All these genes are associated with psychiatric conditions, ranging from ID i.e., *CNKSR2* (Kessels and Malinow, 2009; Vaags et al., 2014), *CTNNB1* (Tucci et al., 2014), *ANK3* (Iqbal et al., 2013), *CASK* (Tarpey et al., 2009) and ASD i.e., *CTNND2* (Turner et al., 2015), *ANK3* (Iqbal et al., 2013) to schizophrenia i.e., *CNKSR2* (GWAS, PGC-SCZ 2014) and bipolar disorder ANK3 (Ferreira et al., 2008).

Similarly, the impairment in LTP and LTD and the deficits in memory and learning observed in the p140Cap KO mice (Repetto et al., 2014) could be sustained by improper functioning and/or localisation of several of these newly identified p140Cap interactors, such as the NMDARs subunits, the AMPA receptors, PSD-95, and the Shank family. *GRIN1, GRIN2A*, and *GRIN2B* are key in LTP induction, synaptic plasticity, learning and memory (Bliss and Collingridge, 1993; Bliss et al., 2014; Shipton and Paulsen, 2014). Synaptic trafficking of AMPARs is necessary for the induction of LTP, and GluR1-deficient mice exhibit impaired hippocampus-dependent spatial working memory and one-trial spatial memory (Kessels and Malinow, 2009). In mice lacking PSD-95, NMDA-dependent LTP and LTD frequency is shifted to enhanced LTP, consistent with severely impaired spatial learning (Migaud et al., 1998). Mice mutant for Shank proteins exhibit a large spectrum of defects in LTP and LTD (Yoo et al., 2014). Identifying the molecular complexes controlling forms of plasticity that underlie memory consolidation and retrieval has direct implications for ID and associated co-morbidities, including ASD and ADHD. It is also important for psychiatric disorders with a cognitive impairment component and/or with a premorbid cognitive deficit, as documented in schizophrenia (Reichenberg et al., 2010).

As mentioned above, *SRCIN1* has not been genetically associated with psychiatric disorders. A *de novo* missense variant resulting in a p.Glu912Asp change predicted to be probably damaging by the bioinformatic predictor Polyphen-2 and not detected in the 60,706 unrelated controls of the Exome Aggregation Database (ExAC) was found in a schizophrenia cohort (Fromer et al., 2014). Further, based on the data in ExAC, *SRCIN1* is significantly depleted in both loss-of-function and missense variants, indicating that the gene is highly intolerant to detrimental variation and so likely a source of disease risk if mutated.

In conclusion, our findings place p140Cap at the crossroad of a highly susceptible synaptic network disrupted in psychiatric and neurological disorders. These findings reinforce the current hypothesis that synaptic dysfunction is a component of all these disorders and have repercussions on our understanding of the underpinnings of psychiatric disorders and their shared comorbidity.

## Ethics statement

This study was carried out in accordance with the recommendations of Italian Ministry of Health. The protocol was approved by the Their Commitee with the number 49/2014-PR.

## Author contributions

AAl, CA, IR, and AM, design, collection and assembly of data, data analysis and interpretation; AAd, YC, and EB collection and analysis of MS data; OS, JA, CM, UA, SD, JB, and AB data analysis and interpretation; AAl, OS, JA, SD, EM. MM, ET, and PD design, interpretation of the data and manuscript writing. All the authors contributed to draft the work or revise it critically for important intellectual content. All the authors gave a final approval of the version to be published. All the authors agree be accountable for all aspects of the work in ensuring that questions related to the accuracy or integrity of any part of the work are appropriately investigated and resolved.

### Conflict of interest statement

The authors declare that the research was conducted in the absence of any commercial or financial relationships that could be construed as a potential conflict of interest.
