# Supplementary figures and images for: Synaptic Interactome Mining Reveals p140Cap as a New Hub for PSD Proteins Involved in Psychiatric and Neurological Disorders
Source: Front Mol Neurosci. 2017 Jun 30;10:212. doi: 10.3389/fnmol.2017.00212 (PMC5492163; doi:10.3389/fnmol.2017.00212)

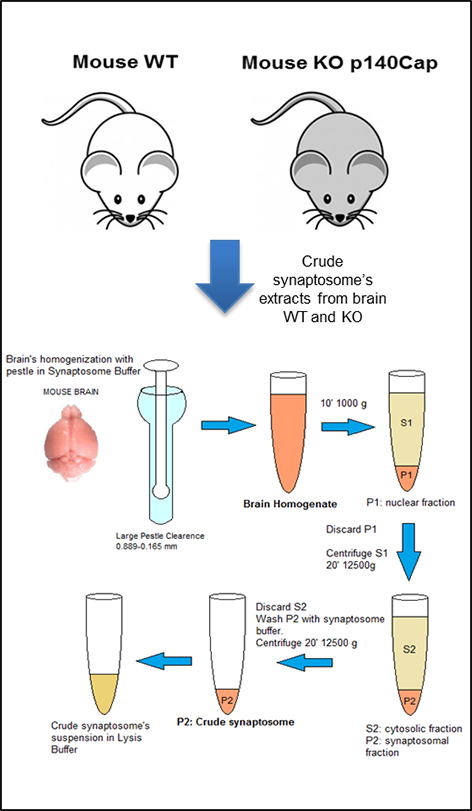

Supplement: Supplementary file 10 [file Image1.TIF]

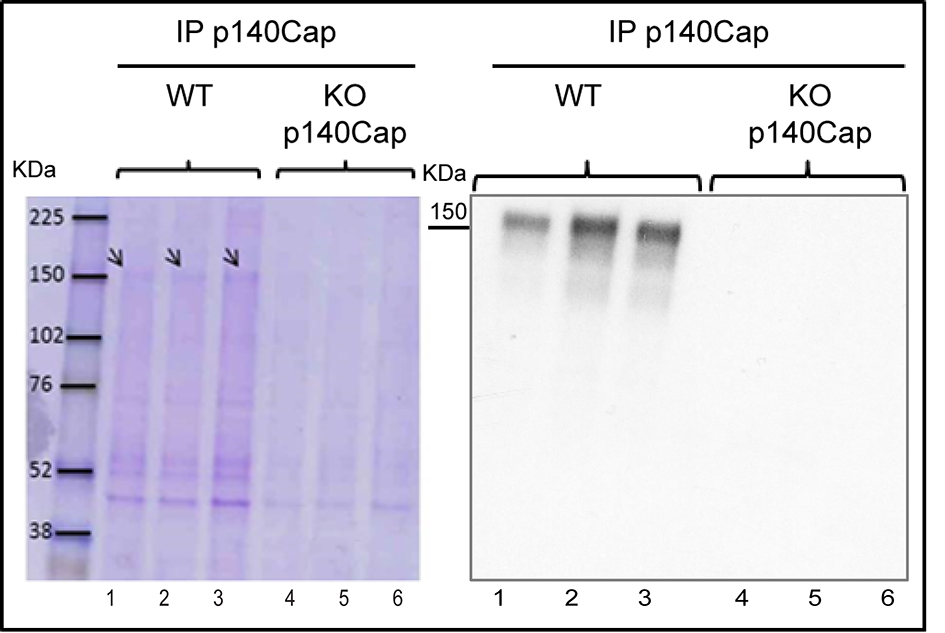

Supplement: Supplementary file 11 [file Image2.TIF]

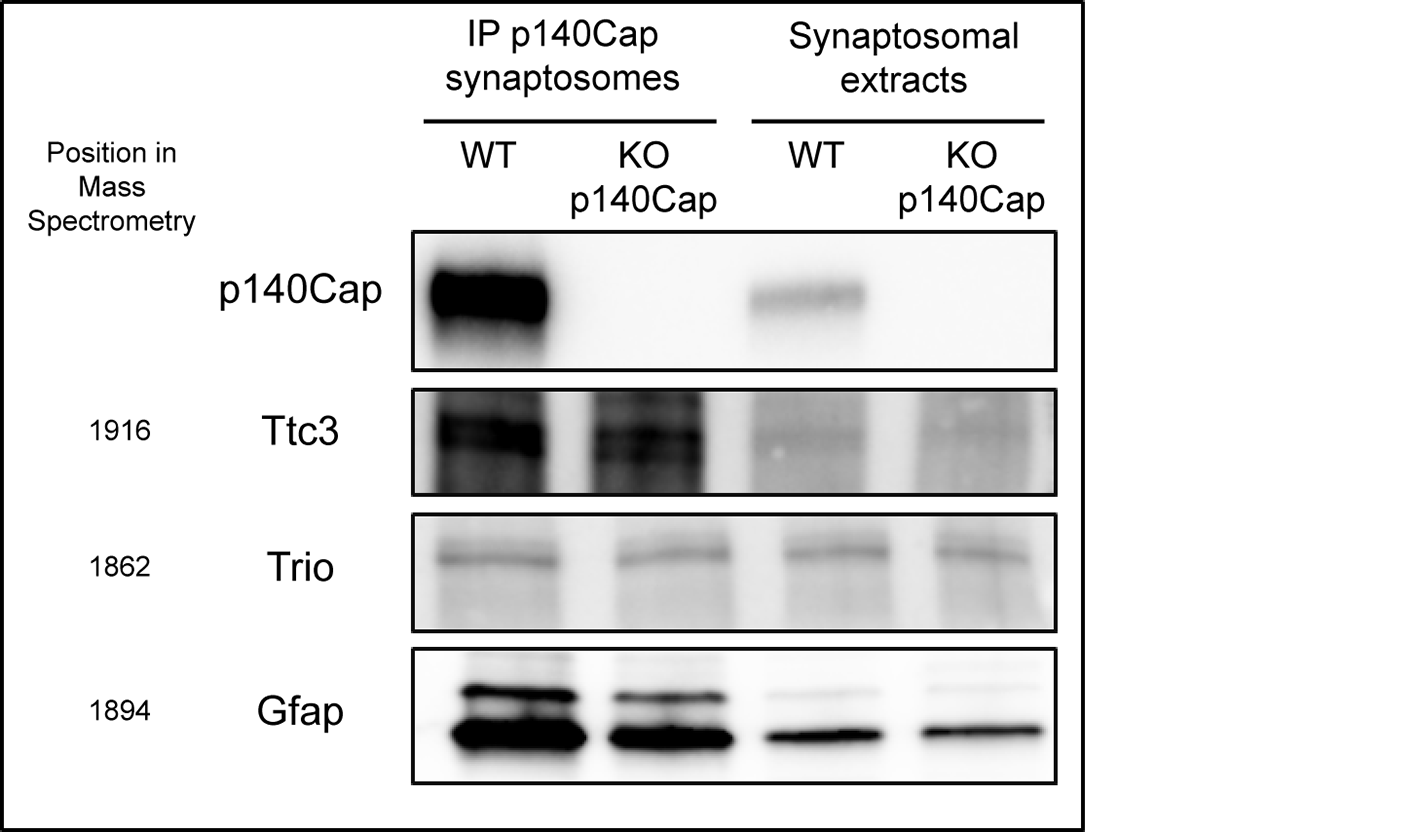

Supplement: Supplementary file 12 [file Image3.TIF]
